# Synchronous disease onset and flares in siblings with PFAPA

**DOI:** 10.1186/s12969-022-00744-0

**Published:** 2022-10-05

**Authors:** Kristen L. Dammeyer, Amanda Schneider, Max M. April, Philip J. Kahn

**Affiliations:** 1 New York University Grossman School of Medicine, New York, NY USA; 2 Department of Pediatrics, Hassenfeld Children’s Hospital at NYU Langone Medical Center, 160 E 32nd Street, L3 Medical, 10016 New York, NY USA; 3 Department of Otolaryngology Head and Neck Surgery, New York University Grossman School of Medicine, New York, NY USA

**Keywords:** Autoinflammatory, Inflammation, Fever, Periodic fever, Tonsillectomy

## Abstract

**Background:**

Periodic fever, aphthous stomatitis, pharyngitis, and cervical adenitis (PFAPA) is a clinical syndrome of unclear etiology. PFAPA has generally been considered a non-hereditary fever syndrome; however, this has been called into question with recent reports of family clustering. Few reports have been published describing siblings with PFAPA. To our knowledge, this is the first report of siblings with near simultaneous onset of disease followed by synchronous disease flares.

**Case Presentation:**

We describe the case of near simultaneous onset of periodic fever, aphthous stomatitis, pharyngitis, and cervical adenitis in siblings followed by synchronous disease flares of clear frequency and nearly identical character. Flares were characterized predominantly by fever, aphthous ulceration, cervical lymphadenitis, and the absence of infection. The fever episodes demonstrated a robust response to glucocorticoids and recurred in the same staggered manner every four weeks, with complete absence of symptoms and normal growth and development between episodes. Nine months after onset, the older sibling, a 5-year-old female, underwent tonsillectomy resulting in dramatic resolution of episodes. At the same time, her 2-year-old sister experienced resolution of her fever episodes, though she did not undergo tonsillectomy herself.

**Conclusion:**

This is an unusual case of simultaneous onset PFAPA followed by synchronous disease flares. PFAPA is an uncommon clinical syndrome, and it is rarely diagnosed in siblings. The etiology of PFAPA remains unclear. Though the disease is classically considered sporadic, there is a growing body of evidence to suggest that PFAPA may be heritable.

## Background

Periodic fever, aphthous stomatitis, pharyngitis, and cervical adenitis syndrome (PFAPA) was first described in 1987[1]. The diagnosis of PFAPA is clinical, requiring the presence of characteristic features and the exclusion of alternative causes. Accepted diagnostic criteria include [1] regularly occurring fevers with an early age of onset [2] symptoms in the absence of upper respiratory tract infection with at least one of the following clinical signs: aphthous stomatitis, cervical lymphadenitis, and pharyngitis, [3] exclusion of cyclic neutropenia, [4] complete absence of symptoms between episodes, and [5] normal growth and development [2]. Updated clinical classification criteria were established in 2019 requiring patients to fulfill at least seven out of the following 8 manifestations including the presence of pharyngitis, 3–6 day duration of episodes, cervical adenitis, and periodicity as well as the absence of diarrhea, chest pain, skin rash and arthritis [3]. The etiology of PFAPA syndrome remains unclear, though it is generally thought to be the result of immune dysregulation. It is considered a non-hereditary disease; however, this has been the subject of debate in recent literature [4]. PFAPA syndrome is self-limited with a favorable prognosis. Symptomatic treatment can be achieved with oral glucocorticoids, which result in dramatic resolution of fever, but do not prevent recurrence. In most children, fever episodes become less frequent over time and eventually cease altogether. Tonsillectomy is an effective option for decreasing the frequency and severity of episodes with a significant potential for cure [5].

## Case Presentation

Here, we report the case of two siblings with PFAPA who experienced near simultaneous onset of disease and, subsequently, synchronized fever episodes. Patient A is a 5-year-old female presenting with fever occurring every 4 weeks, lasting 5 days with a maximum temperature of 38.9 °C. She was noted to have aphthous ulceration, pharyngeal erythema, enlarged tonsils, and enlarged cervical lymph nodes when febrile. She would also report sore throat, mild abdominal pain, tiredness and slightly decreased oral intake. She was otherwise asymptomatic between episodes with normal growth and development.

Five days after the onset of fever in Patient A, her 2-year-old sister (Patient B) began experiencing fever occurring every 4 weeks, lasting 5 days with a maximum temperature of 40.0 °C. Aphthous ulceration and enlarged cervical lymph nodes were also noted. She was otherwise asymptomatic between episodes with normal growth and development. The episodes followed a consistent pattern such that Patient B experienced onset of each fever five days after fever onset in Patient A.

Episodes in both patients were characterized by elevated acute phase reactants, with the absence of neutropenia. During an acute disease flare, erythrocyte sedimentation rate was elevated to 82 mm/h in Patient A and 120 mm/h in Patient B (range 0–20 mm/h), while C-reactive protein was elevated to 48.6 mg/L in Patient A and 226 mg/L in Patient B (range 0–5 mg/L). Workup by infectious diseases, hematology, and oncology was unremarkable and included CBC, inflammatory markers, and throat culture. Patient A’s workup also included a comprehensive metabolic panel, quantitative immunoglobulins, Epstein-Barr Virus quantitative PCR and serology, and Borrelia PCR. Both patients demonstrated a robust response to prednisolone, with complete resolution of fever within hours.

Nine months after onset, the family opted for tonsillectomy and adenoidectomy for Patient A given her older age, resulting in dramatic resolution of her fever episodes, which have not recurred at nine-month follow-up. Shortly after Patient A’s tonsillectomy, Patient B experienced two fevers inconsistent with her typical episodes, as she had rhinorrhea and cough raising greater suspicion for viral syndrome. Otherwise, she has had no recurrence of fever. Of note, the patients’ father and paternal aunt experienced recurrent pharyngitis, which resolved following tonsillectomy in childhood. The father is of Chinese and Srilankan ancestry, and the mother is of European Ashkenazi Jewish ancestry.

## Discussion and conclusions

Autoinflammatory fever syndromes are often the result of a genetic defect; however, PFAPA has classically been considered a non-hereditary fever syndrome. The concept that PFAPA is sporadic rather than hereditary has been called into question with recent literature reporting that a proportion of PFAPA patients have a family history of recurrent fever, aphthous stomatitis, or pharyngitis which may represent reduced penetrance of the disease and undiagnosed PFAPA [6, 7]. Though family clustering may be seen, a single causative gene has not been identified, with speculation that PFAPA is the result of either mutations in a small number of genes or the combined influences of multiple genetic mutations and gene-environment interactions [4].

Few reports have been published describing siblings with the syndrome of PFAPA [8–11]. To our knowledge, this is the first report of siblings with near simultaneous onset of disease. There was no overt infectious trigger reported by the family, but notably, onset of disease occurred while the family was quarantining during the height of the COVID-19 pandemic in New York. Even more intriguing is the synchronicity of fever flare pattern and symptoms. Furthermore, the elder sister underwent tonsillectomy, which was ultimately curative. Interestingly, the younger sister’s fever episodes have subsided following her sister’s tonsillectomy, possibly suggesting that their flares were indeed interrelated, potentially via shared environmental triggers. Periodic fevers appear to run on the paternal side of this family, perhaps with variable penetrance of the complete PFAPA phenotype. It remains unclear how and why the siblings experienced simultaneous onset of synchronized recurrent fever. Further study is warranted.

## Data Availability

Patient data used during this report are available from the corresponding author on reasonable request.

## Declarations

### Author’s information

KD – Medical student, NYU Grossman School of Medicine, New York, NY.

AS – Pediatric Allergist and Immunologist, Hassenfeld Children’s Hospital, New York, NY.

MA – Pediatric Otolaryngologist, Hassenfeld Children’s Hospital, New York, NY.

PK – Pediatric Rheumatologist, Hassenfeld Children’s Hospital, New York, NY.

## List of abbreviations

PFAPA: Periodic fever, aphthous stomatitis pharyngitis and cervical adenitis.
